# Plant Growth Promoting Rhizobacteria Alleviate Aluminum Toxicity and Ginger Bacterial Wilt in Acidic Continuous Cropping Soil

**DOI:** 10.3389/fmicb.2020.569512

**Published:** 2020-11-30

**Authors:** Shuting Zhang, Qipeng Jiang, Xiaojiao Liu, Liehua Liu, Wei Ding

**Affiliations:** College of Plant Protection, Southwest University, Chongqing, China

**Keywords:** continuous cropping, aluminum toxicity, extremely acidic soil, ginger bacterial wilt, plant growth promoting rhizobacteria (PGPR)

## Abstract

Long-term monoculture cropping is usually accompanied by soil acidification and microbial community shifts. Soil aluminum ions are dissolved under acidic condition (pH < 5.0), and the resulting aluminum bioavailability can cause toxic effects in plants. In this study, we investigated the bacterial community compositions and aluminum toxicity in fields monocultured with ginger for 35 years, 15 years, and 1 year. Within these fields are ginger plants without and with ginger bacterial wilt disease. The results confirmed that the degree of aluminum toxicity in the diseased soil was more severe than that in the healthy soil. Continuous cropping can significantly increase the bacterial diversity and change the bacterial community composition of ginger rhizosphere soil. The relative abundance of plant growth-promoting rhizobacteria (PGPRs) was increased in the soils used for the continuous cropping of ginger. Additionally, aluminum toxicity had a significant positive correlation with *Bacillus, Pseudomonas, Arthrobacter*, and *Serratia* in healthy soils. Based on these results, aluminum stress may stimulate the increase of PGPRs (*Bacillus, Pseudomonas, Arthrobacter*, and *Serratia*), thereby alleviating ginger aluminum toxicity and bacterial wilt in extremely acidic soil (pH < 4.5).

## Introduction

Continuous cropping refers to growing the same crop in the same soil year after year (Shipton, 1977). Long-term continuous cropping usually enriches soil-borne pathogens, leading to yield declines (Liu et al., 2014b). Continuous cropping is also closely related to the occurrence of bacterial wilt (She et al., 2016).

Soil acidification is a serious global environmental problem of economic concern that limits the sustainable development of modern agriculture, and soil acidification (pH 4.5–5.5) aggravates the occurrence of tobacco bacterial wilt (Li et al., 2017; Singh et al., 2017). At slightly acidic or neutral soil pH values, Aluminum (Al) occurs primarily in the form of insoluble aluminosilicates or oxides. With the decrease of the pH, dissolved aluminum increased in the soil (Kochian, 1995). The effect of the pH on the dissolution of aluminum varies with the soil type. At pH values below 5, large amounts of aluminum ions are dissolved from clay minerals (Baba and Okazaki, 2000; Kochian et al., 2015). When pH values are below 4.5, the dissolution of aluminum in three acidic soils followed the order of red soil > lateritic red soil > latosol (Xu and Ji, 1998). Aluminum has a wide range of toxic effects on plants. Aluminum ions first inhibit root cell expansion and elongation; however, over the longer term, cell division is also inhibited, thereby inhibiting root growth (Kochian et al., 2015). Moreover, aluminum can alter the usual functions of the plasma membrane by inducing membrane lipid peroxidation (Massot et al., 1999; Vitorello et al., 2005), inducing imbalances in the uptake and acquisition of various mineral elements, such as Ca, Mg, and P (Olivares et al., 2009; Silva et al., 2012). Aluminum (Al) toxicity is a primary factor in the reduction of crop yields in acidic soils (Kochian et al., 2015).

There are many forms of aluminum in acidic soils, and the toxic effects of the different forms of Al (speciation) on plant growth diminish in the following order: Al^3+^, Al(OH)^2+^, ${\text{Al(OH)}}_{2}^{+}$, and ${\text{Al(OH)}}_{4}^{-}$ (Bojorquez-Quintal et al., 2017). However, Al^3+^ is considered the most toxic to plant among all the forms of aluminum (Schmitt et al., 2016). Therefore, the concentration of aluminum ions is an important indicator for evaluating plant aluminum toxicity (Rehmus et al., 2014). Soil acidification causes pronounced changes in the soil chemistry, such as the dissolution and release of Al, Mn, and Fe, the depletion of Mg^2+^ and Ca^2+^ and deficiencies of phosphorous (Kochian et al., 2004). During the decomposition process of acidic organic groups in litter, ecologically and physiologically essential Ca^2+^ or acidic cations such as Al^3+^ and Fe^3+^ show signs of toxicity (Luo, 2000). Therefore, using Ca^2+^/Al^3+^, Mg^2+^/Al^3+^, and Ca^2+^/(Ca^2+^+Fe^3+^+Al^3+^) to assess aluminum toxicity is better than only using the absolute concentrations of Al^3+^ (Kazda and Zvacek, 1989; Wit et al., 2001; Liu et al., 2014a).

Microorganisms play an indispensable role in maintaining soil health (Bruggen and Semenov, 2000). Continuous cropping influences tobacco bacterial wilt by affecting the soil microbial community composition and structure (She et al., 2016). The soil pH is the main predictor of soil bacterial community changes, and the soil pH has a strong influence on the relative abundance and diversity of bacteria (Rousk et al., 2010; Zhalnina et al., 2015). However, the role of aluminum ions in influencing the microbial community composition in acidic soil is still unclear.

In response to plant stresses, many microorganisms increase in number or proportion (Liu et al., 2020a), as is the case for plant growth-promoting rhizobacteria (PGPRs), which have the potential to improve crop production under different stress conditions alone and/or in combination with other microbes (Nadeem et al., 2015). Under stress conditions, PGPRs are beneficial through particular mechanisms, such as regulating nutrient uptake (Raj et al., 2003), inducing systemic resistance (Choudhary et al., 2007), and preventing disease (Guo et al., 2004) and aluminum stress (Labanca et al., 2020; Shi et al., 2020). Some of these PGPRs belong to important genera, including *Bacillus, Paenibacillus, Pseudomonas, Serratia, Arthrobacter, Azospirillum, Burkholderia, Streptomyces*, and *Flavobacterium*, among others (Lugtenberg and Kamilova, 2009; Niu et al., 2011; Nadeem et al., 2015). Strains belonging to *Bacillus, Pseudomonas, Streptomyces*, and *Serratia* have been used as PGPRs for ginger growth promotion and biocontrol studies (Tahat and Kamaruzaman, 2010; Dinesh et al., 2015).

Ginger (*Zingiber officinale* Roscoe) is one of the most economically valuable plants and is used worldwide as a spice and flavoring agent (Li et al., 2018). Ginger bacterial wilt, which is caused by *Ralstonia solanacearum*, is an important soil-borne disease of ginger (Ming et al., 2005). Ginger is an extremely acid-tolerant species with a minimum pH tolerance of 3.3 (Islam et al., 1980). Soil with a pH value below 4.5 is considered extremely acidic soil (Zhou et al., 2011). According to our survey, in Rongchang, Chongqing, ginger is planted in a continuous mono-cropping pattern, and the soil is extremely acidic (pH < 4.5). Ginger bacterial wilt is a widespread soil-borne disease in this area. Nonetheless, the ginger in some areas is still healthy after continuous mono-cropping for over 15 years. In this study, we collected soil without bacterial wilt, which was referred to as “healthy” soil, from areas with 35 years (35H), 15 years (15H), and 1 year (1H) of continuous cropping. The soil with bacterial wilt was referred to as “diseased” soil and was collected from areas with 35 years (35D) and 15 years (15D) of continuous cropping. We analyzed the index of aluminum toxicity values and the bacterial community composition of the samples. We hypothesized that (i) aluminum ions influence the occurrence of ginger bacterial wilt under extreme acidification conditions and (ii) PGPRs, such as *Bacillus* and *Pseudomonas*, play an important role in overcoming continuous mono-cropping obstacles under aluminum toxicity stress. Specifically, this study aimed to determine the reason why ginger bacterial wilt varies in occurrence in extremely acidic continuous cropping soil.

## Materials and Methods

### Soil Sampling

Soil sample were collected from an agricultural field site owned by the Changling farm in Rongchang, Chongqing city, China (29°30′6.50″ N, 105°22′41.13″ E). The tested soil has been classified as clay. According to the survey, the incidence of bacterial wilt with 35 and 15 years of continuous cropping was 5 and 53%, respectively. We chose the long-term continuous monoculture fields with ginger wilt as diseased soil (continuous cropping for 35 years, 35D, and continuous cropping for 15 years, 15D), whereas sites without disease were treated as healthy soil (continuous cropping for 35 years, 35H, continuous cropping for 15 years, 15H, and 1 year, 1H). The 35H, 35D, 15H, and 15D soils were collected in August 2014. The 1H soil was collected in June 2015. The distance between each sampling point was within 100 m, and the ecological environment was consistent. We pulled out the ginger plants and then collected the soil attached to the roots as the rhizosphere soil. Five ginger plants were randomly selected, and the rhizosphere soil was collected and mixed into one sample as a replicate, with three replicates collected for each treatment. The soil samples were transported to the laboratory at 4°C and then stored at −40°C before DNA extraction.

### Soil Chemical Properties

The soil pH was measured at a soil/water ratio of 1/2.5 (weight/volume). The air-dried soil (10 g, 2 mm) and 25 mL of deionized water were shaken together for 5 min and left to settle for 30 min, after which the pH was determined with a pH electrode (InLab Science, Mettler Toledo, Switzerland) (Pietri and Brookes, 2008). The soil exchangeable aluminum ions were extracted with 1 mol/L KCl and then calibrated with 0.02 mol/L NaOH. The available iron, exchangeable magnesium, exchangeable calcium, and other basic chemical properties of each soil sample were determined using standard methods in the soil analysis laboratory of Southwest University, China (Supplementary Table 1). The aluminum ion concentration, Ca^2+^/Al^3+^, Mg^2+^/Al^3+^, and Ca^2+^/(Ca^2+^+Fe^3+^+Al^3+^) were used as indicators for evaluating aluminum toxicity (Kazda and Zvacek, 1989; Wit et al., 2001; Liu et al., 2014a; Rehmus et al., 2014).

### DNA Extraction and Sequencing Library Construction

The total genomic DNA was extracted from 0.5 g of soil using the Omega Biotek Soil DNA kit (Omega Biotek, USA), by following the standard protocol. PCR amplifications were conducted with 338 forward primers (5′-ACTCCTACGGGAGGCAGCAG-3′) and 806 reverse primers (5′-GGACTACHVGGGTWTCTAAT-3′), which amplified the V3–V4 region of the 16S rRNA gene (Xu et al., 2016).

The PCR amplification and sequencing library construction were performed in accordance with the procedures described by Zhang S. et al. (2017). The raw sequence analysis was performed using the Illumina MiSeq paired end 250 bp protocol (Illumina, Inc., San Diego, CA, USA) at the Center for Genomic Research, Shanghai Majorbio Biotechnology Co. Ltd, China. In total, 394,283 sequences were obtained from the 15 samples through 16S rRNA high-throughput sequencing analysis. The average read length was 434 bp. The raw reads were deposited into the NCBI short-reads archive database under accession number SRP224758.

### Statistical Analysis

The Raw Illumina FASTQ files were processed in QIIME v1.7.0 (Quantitative Insights Into Microbial Ecology) (Caporaso et al., 2010). The operational taxonomic units (OTUs) were identified based on a threshold of 97% pairwise identity by QIIME.

The bacterial community data was analyzed on the free online Majorbio Cloud Platform (http://www.i-sanger.com/). To examine the effects of the number of continuous cropping years and disease on the microbial community structures, non-metric multidimensional scaling (NMDS) and PERMANOVA (Permutational Multivariate Analysis Of Variance) based on the unweighted UniFrac distance were conducted with corresponding function in the “vegan” package in the R environment. The selection of PGPRs referred to Nadeem et al. (2015). The relative abundances of PGPRs were multiplied by 100,000 and then LOG10 values were calculated. The figures were created using Origin 9.0 software and GraphPad Prism 8.0.1. The mean and standard error for each set of data were calculated by one-way analysis of variance (ANOVA) with Tukey's honest significant difference test (*P* < 0.05) using SPSS software (version 17.0). The correlations between aluminum ions, Ca^2+^/Al^3+^, Mg^2+^/Al^3+^ and Ca^2+^/(Ca^2+^+Fe^3+^+Al^3+^), and PGPRs were analyzed by Spearman analysis using SPSS software (version 17.0).

## Results

### Aluminum Toxicity Was More Severe in Diseased Soil

The pH values of all the soil samples were below 4.5, with that of 15D being the highest (pH = 4.41) and that of 35H being the lowest (pH = 3.96). With the increase in continuous cropping years, the soil pH decreased. Additionally, the concentrations of exchangeable aluminum and available iron significantly increased; however, the concentrations of exchangeable magnesium and exchangeable calcium and the rates of Ca^2+^/Al^3+^, Mg^2+^/Al^3+^, and Ca^2+^/(Ca^2+^+Fe^3+^+Al^3+^) significantly decreased (*P* < 0.05). The concentration of exchangeable magnesium and exchangeable calcium and the rate of Ca^2+^/Al^3+^, Mg^2+^/Al^3+^, and Ca^2+^/(Ca^2+^+Fe^3+^+Al^3+^) in healthy soil were significantly higher than those in diseased soil (*P* < 0.05, Table 1).

**Table 1:** Aluminum toxicity index of different samples.

**Treatment**	**pH**	**ExAl (mg/kg)**	**AvaFe (mg/kg)**	**ExMg**	**ExCa (mg/kg)**	**Ca^**2+**^/Al^**3**^**	**Mg^**2+**^/Al^**3+**^**	**Ca^**2+**^/**
				**(mg/kg)**				**(Ca^2+^+**Fe**^3+^+**
								**Al^**3+**^)**
35H	3.96 ± 0.02a	438.02 ± 24.01c	42.04 ± 0.05d	155.50 ± 2.28b	454.37 ± 89.66b	1.03 ± 0.19ab	0.36 ± 0.02b	0.48 ± 0.04b
35D	4.05 ± 0.01ab	385.00 ± 16.77bc	42.88 ± 0.06e	109.62 ± 3.64a	185.12 ± 8.12a	0.48 ± 0.03a	0.29 ± 0.02a	0.30 ± 0.01a
15H	4.22 ± 0.01bc	337.30 ± 10.17ab	39.85 ± 0.06b	374.37 ± 4.05c	1184.12 ± 74.42c	3.50 ± 0.12c	1.11 ± 0.03d	0.76 ± 0.01d
15D	4.41 ± 0.00c	299.05 ± 0.80a	40.86 ± 0.05c	191.25 ± 8.54b	526.87 ± 14.19b	1.76 ± 0.05b	0.64 ± 0.03c	0.61 ± 0.01c
1H	4.26 ± 0.10bc	334.09 ± 23.86ab	6.32 ± 0.36a	394.67 ± 13.82c	1949.96 ± 31.33d	5.89 ± 0.36d	1.19 ± 0.08d	0.85 ± 0.01d

*The results are the mean of three measurement replicates ± standard error. Small letters indicate a significant difference among different samples (one-way ANOVA, P < 0.05). ExAl, exchangeable aluminum; AvaFe, available iron; ExMg, exchangeable magnesium; ExCa, Exchangeable calcium*.

### Continuous Cropping Changed the Bacterial Community Structure

The rarefaction curves are illustrated in Supplementary Figure 1. The continuous-cropping samples showed increased Shannon index values and significantly reduced Simpson values (*P* < 0.05, Supplementary Figure 2). This indicated that continuous cropping significantly increased the diversity of soil microbes. There was no significant difference in the microbial richness among the different treatments (Supplementary Figure 2).

**Figure 1:**
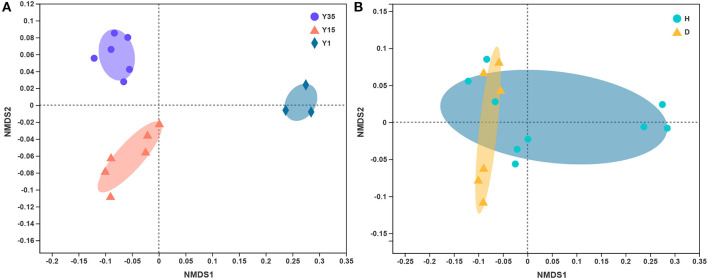
Non-metric multidimensional scaling (NMDS) of the unweighted UniFrac analysis based on the OTU level. **(A)** Grouped by continuous cropping years (pseudo-F: 7.005, *R*^2^ = 0.539, *P* = 0.001, PERMANOVA). **(B)** Grouped by healthy and diseased. (pseudo-F: 2.491, *R*^2^ = 0.161, *P* = 0.038, PERMANOVA). Y35—continuous cropping for 35 years, Y15—continuous cropping for 15 years, Y1—one year; H—the heathy soil without bacterial wilt, D—diseased soil with bacterial wilt.

The composition of the bacterial community changed significantly among different continuous cropping years, as visualized in the NMDS ordination (Figure 1A), resulting in a two-dimensional final solution with a stress value of 0.056. The PERMANOVA analysis revealed that the difference was statistically supported (*P*-value = 0.001, pseudo-F = 22.147, R^2^ = 0.788). However, there was no significant distinction between the diseased and healthy soil (*P*-value = 0.073, pseudo-F = 2.413, R^2^ = 0.156, using PERMANOVA, Figure 1B).

**Figure 2:**
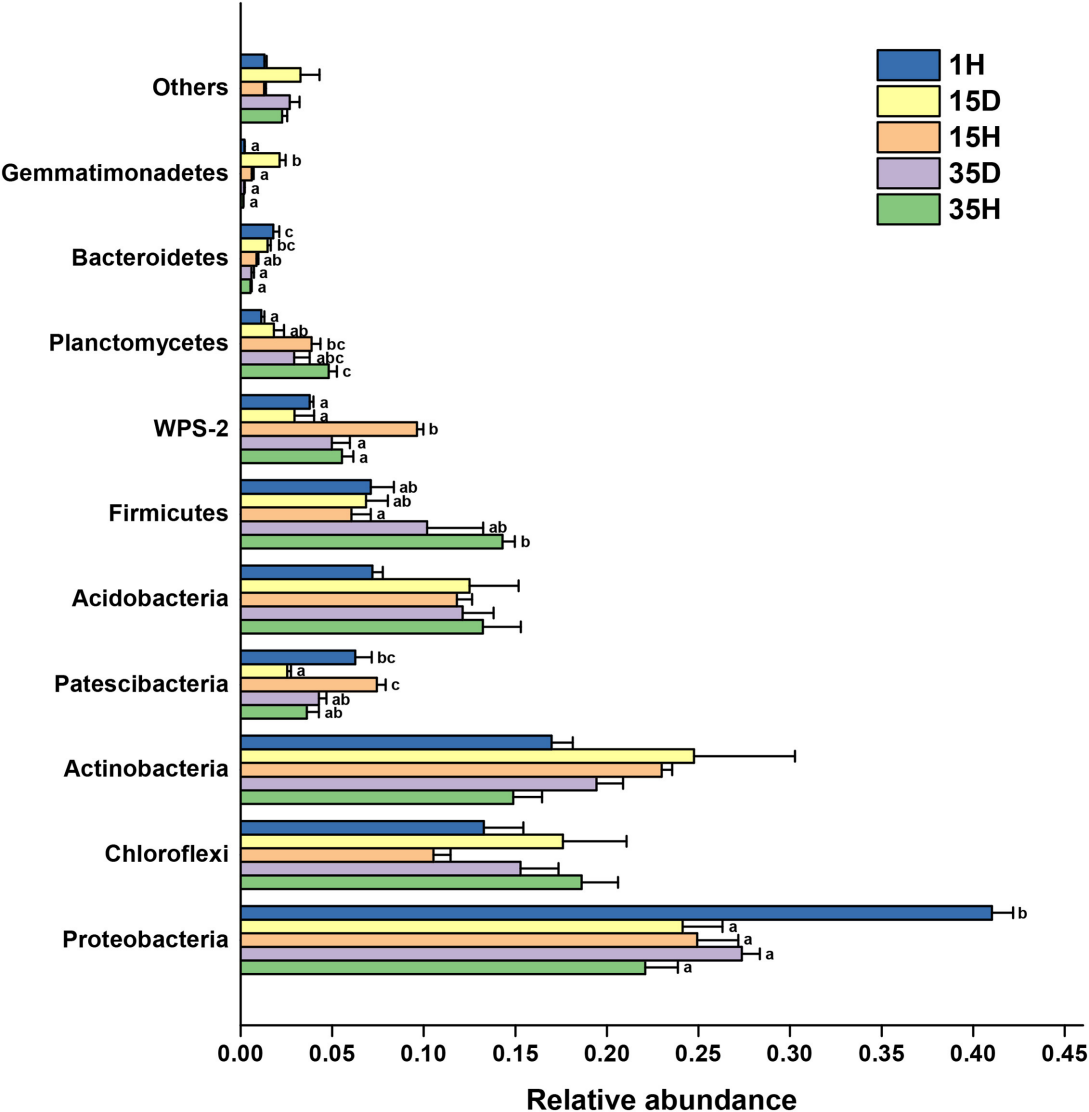
The relative abundance of the main phyla in the soil samples (*n* = 3). Different letters indicate significant differences based on one-way ANOVA (Turkey test) at *P* < 0.05.

The distribution of the bacterial groups at the phylum level is shown in Figure 2. The relative abundance of Proteobacteria in 1H was significantly higher (*P* < 0.05) than those in the other groups. Upon comparing 35H with 1H, the Planctomycetes group was significantly higher, while the Bacteroidetes was significantly lower (*P* < 0.05). The relative abundances of Patescibacteria and WPS-2 in 15H were significantly higher than those in 15D. However, the trend of the Gemmatimonadetes abundance was the exact opposite. There was no significant difference in the bacterial community composition at the phylum level between the diseased and healthy soil after 35 years of continuous cropping. The relative abundance of *Ralstonia*, which the genus of the ginger bacterial wilt pathogen, was higher in diseased soil than in healthy soil (Supplementary Figure 3). Among the samples, 15D had the highest relative abundance of *Ralstonia*, which was significantly higher than those in 1H (*P* = 0.0017), 15H (*P* = 0.0154), and 35H (*P* = 0.0028), respectively.

### Aluminum Stress Stimulated Plant Growth-Promoting Rhizobacteria Enrichment

**Figure 3:**
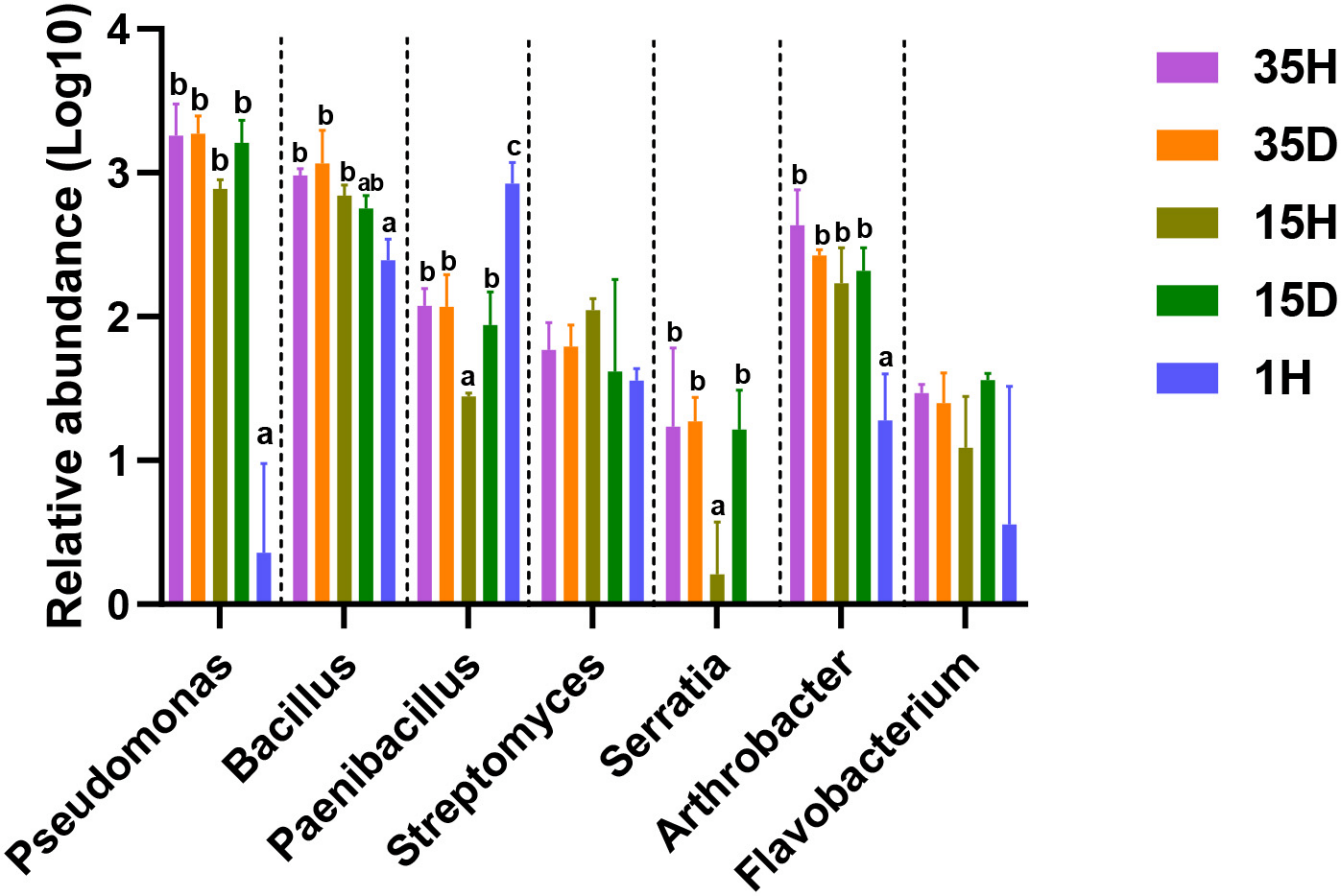
The relative abundance of plant growth-promoting rhizobacteria (PGPRs). The relative abundance of each PGPRs was multiplied by 100,000, and then the LOG10 value was calculated. Different letters indicate significant differences based on one-way ANOVA (Turkey test) at *P* < 0.05.

Compared with 1H, the relative abundance of PGPRs, such as *Pseudomonas, Bacillus, Serratia*, and *Arthrobacter*, was increased in the rhizosphere soils of long-term continuous-cropping areas (Figure 3). There was a significant decrease in the relative abundance of *Paenibacillus* after long-term continuous cropping. Interestingly, *Bacillus* had an extremely significantly positive correlation (Spearman, *r* = 0.670, *P* = 0.006) with aluminum ions in all soil samples. However, Ca^2+^/Al^3+^, Mg^2+^/Al^3+^, and Ca^2+^/(Ca^2+^+Fe^3+^+Al^3+^) had extremely significantly negative correlation with *Pseudomonas, Bacillus, Arthrobacter*, and *Serratia* (Supplementary Table 2). Furthermore, aluminum ions had significantly positive correlations with *Pseudomonas* and *Bacillus* in healthy soils. Ca^2+^/Al^3+^, Mg^2+^/Al^3+^, and Ca^2+^/(Ca^2+^+Fe^3+^+Al^3+^) had extremely significantly negative correlations with *Pseudomonas, Bacillus, Arthrobacter*, and *Serratia* in healthy soil (Table 2).

**Table 2:** Correlation coefficients (Spearman) between aluminum toxicity index and the PGPRs in healthy and diseased soil.

**PGPR**	**Soils**	**ExAl**	**Ca^**2+**^/Al^**3**^**	**Mg^**2+**^/Al^**3+**^**	**Ca^**2+**^/ (Ca**^2+^+**Fe**^3+^+**Al^**3+**^)**
*Pseudomonas*	H	0.787^*^	−0.946^**^	−0.912^**^	−0.946^**^
	D	−0.058	0.257	0.029	0.257
*Bacillus*	H	0.783^*^	−0.933^**^	−0.900^**^	−0.933^**^
	D	0.638	−0.543	−0.600	−0.543
*Paenibacillus*	H	−0.033	0.350	0.367	0.350
	D	−0.029	0.086	−0.029	0.086
*Streptomyces*	H	−0.133	−0.483	−0.167	−0.483
	D	−0.261	0.371	0.200	0.371
*Arthrobacter*	H	0.550	−0.900^**^	−0.733^**^	−0.900^**^
	D	0.290	−0.143	−0.200	−0.143
*Serratia*	H	0.639	−0.913^**^	−0.694^*^	−0.913^**^
	D	−0.290	0.029	0.257	0.029
*Flavobacterium*	H	0.025	−0.310	−0.167	−0.310
	D	−0.406	0.657	0.429	0.657

H, healthy soils; D, diseased soils.

* Indicated P-values below 0.05,

** *Indicated P-values below 0.01*.

## Discussion

### Continuous Cropping Aggravates the Degree of Aluminum Toxicity and Increases the Occurrence of Bacterial Wilt

The soil pH has a very significant negative correlation with exchangeable aluminum ions (Dong et al., 1999; Xu et al., 2014). By raising the soil pH and Ca content, reducing the aluminum ion concentration can effectively control bacterial wilt (Li and Dong, 2013). In this study, the pH range of the ginger soil sample was between 3.96 and 4.41 (Table 1), which is suitable for the growth of ginger (Islam et al., 1980). As the continuous cropping years increased, Ca^2+^/Al^3+^, Mg^2+^/Al^3+^, and Ca^2+^/(Ca^2+^+Fe^3+^+Al^3+^) in the healthy soils significantly declined. This indicated that the soil aluminum toxicity worsened with the increase in the number of continuous cropping years. Although the concentration of exchangeable aluminum in the healthy soil was higher than that in the diseased soil, the rates of Mg^2+^/Al^3+^, Ca^2+^/Al^3+^, and Ca^2+^/(Ca^2+^+Fe^3+^+Al^3+^) in the healthy soils were also higher than those in the diseased soil. This result indicated that the aluminum toxicity in the diseased soil was more serious than that in the healthy soil. The degree of aluminum toxicity in ginger cropping areas may be a key factor affecting the occurrence of ginger bacterial wilt in extremely acidic soil environments.

### Continuous Cropping Increased the Bacterial Diversity and Changed the Bacterial Community Structure

The bacterial diversity was much higher in the continuous-cropping soils than in 1H (Supplementary Figure 2). This finding was in line with the results of Liu et al. (2020b), who found that soybean continuously cropped for 13 years increased the diversity of soil bacterial communities. An increased soil microbial diversity was found to be responsible for the suppression of soil-borne plant diseases (Mazzola, 2004; Shen et al., 2018). However, in this study, there was no significant difference in bacterial diversity between the diseased and healthy soils. Analysis using PREMANOVA showed that the effect of continuous cropping on the bacterial community structure was stronger than that of the disease factor. This indicated that after long-term continuous ginger mono-cropping, the impact of disease on microorganisms was no longer the main factor.

### Possible Roles of PGPRs in Alleviating Continuous Cropping Obstacles and Aluminum Stress

PGPRs have the potential to ensure plant health under stressful conditions (Nadeem et al., 2015). PGPRs colonize the root surface and rhizospheres, where they compete with pathogenic organisms for nutrients and space (Faina et al., 2010) and/or prevent the proliferation of plant pathogens through the synthesis of different antibiotics (Compant et al., 2005). Multiple *Bacillus* and *Paenibacillus* spp. can promote crop health, suppress plant pathogens by producing antibiotic metabolites, or directly stimulate plant host defenses prior to infection in a variety of ways (Gardener, 2004). *Pseudomonas fluorescens*, which belongs to the genus *Pseudomonas*, is a widely used antagonistic antibacterial agent for controlling bacterial wilt (Anith et al., 2000; Ran et al., 2005), and it can also inhibit the production of soil soluble Al and exchangeable Al (Nkoh et al., 2020). In contrast, *Paenibacillus* is suitable for growth in neutral environments (Liu et al., 2016; Kong et al., 2020) and may possess weak tolerance to aluminum toxicity. She et al. demonstrated that the abundance of *Arthrobacter* had a significant negative correlation with tobacco bacterial wilt disease after long-term continuous cropping (She et al., 2016).

The organic acids released from plant roots, such as malic acid, oxalic acid, and citric acid, can chelate Al ions, reducing the toxicity of Al to plants (Kochian et al., 2015). Malic acid can attract beneficial bacteria such as *Bacillus* to colonize the rhizosphere of plants, thereby enhancing plant resistance (Rudrappa et al., 2008). Al-tolerant bacteria (e.g., *Serratia*) in soil could form Al^3+^-siderophore complexes and promote P uptake to alleviate Al phytotoxicity (Mora et al., 2017). Also, Al-resistant plant cultivars can recruit beneficial microbes to alleviate the stresses (Shi et al., 2020).

Strains belonging to *Bacillus, Pseudomonas*, and *Serratia* have also been used as PGPRs for ginger growth promotion and biocontrol (Dinesh et al., 2015). *Arthrobacter* can both enhance ginger growth and inhibit ginger bacterial wilt (Zhang et al., 2018). *Bacillus* has been extensively studied for growth promotion and the suppression of bacterial wilt caused by *Ralstonia solanacearum* (Hyakumachi et al., 2013; Shiyong et al., 2013). *Bacillus*, as a ginger rhizosphere growth-promoting bacteria, provides effective protection to ginger rhizomes (Jimtha John et al., 2016) and exerts a substantial inhibitory effect on ginger soil-borne diseases, such as rhizome rot (Zhang N. et al., 2017) and bacterial wilt (Yang et al., 2012). In this study, after long-term continuous cropping, the relative abundances of *Pseudomonas, Bacillus, Serratia*, and *Arthrobacter* in the rhizosphere soil of ginger increased significantly (Figure 3). Interestingly, *Bacillus* had an extremely significantly positive correlation (Spearman, *r* = 0.670, *P* = 0.006) with aluminum ions, and Ca^2+^/Al^3+^, Mg^2+^/Al^3+^, and Ca^2+^/(Ca^2+^+Fe^3+^+Al^3+^) had extremely significantly negative correlations with *Pseudomonas, Bacillus, Arthrobacter*, and *Serratia* in all soil samples (Supplementary Table 2). Furthermore, *Pseudomonas* and *Bacillus* were significantly positively correlated with aluminum ions, and Ca^2+^/Al^3+^, Mg^2+^/Al^3+^, and Ca^2+^/(Ca^2+^+Fe^3+^+Al^3+^) had extremely significantly negative correlations with *Pseudomonas, Bacillus, Arthrobacter*, and *Serratia* in the healthy soils (Table 2). These results indicated that the increase of PGPRs, especially *Bacillus, Pseudomonas, Arthrobacter*, and *Serratia*, may be an important factor in relieving continuous cropping obstacles and preventing the occurrence of ginger bacterial wilt under stressful aluminum conditions.

With the increase in continuous cropping years, the soil bacterial community structure changed significantly. The aluminum toxicity of diseased soil was significantly higher than that of healthy soil. In the diseased ginger soil, pH decreased and the aluminum toxicity increased significantly. In the healthy soil with a long continuous cropping duration, the abundance of ginger growth promoting rhizobacteria (PGPRs), such as *Bacillus, Pseudomonas, Arthrobacter*, and *Serratia*, was relatively higher than that of diseased soil, and these potential PGPRs had a significant positive correlation with aluminum toxicity in the healthy soil with a long continuous cropping period. These results indicated that continuous cropping was the main factor affecting the microbial community structure, and aluminum toxicity was a main factor affecting the occurrence of bacterial wilt in extremely acidic soil, and ginger can alleviate aluminum toxicity by stimulating the increase of PGPRs in a long-term aluminum stress environment, thereby reducing the occurrence of ginger bacterial wilt.

## Data Availability Statement

The datasets generated for this study can be found in online repositories. The names of the repository/repositories and accession number(s) can be found in the article/Supplementary Material.

## Author Contributions

WD and SZ designed the experiments. SZ, QJ, and LL performed the experiments and collected the data. SZ and XL analyzed the data. SZ wrote the manuscript. QJ, XL, LL, and WD edited and commented on the manuscript. All authors contributed to the article and approved the submitted version.

## Conflict of Interest

The authors declare that the research was conducted in the absence of any commercial or financial relationships that could be construed as a potential conflict of interest.
