# Supplementary figures and images for: Plant Growth Promoting Rhizobacteria Alleviate Aluminum Toxicity and Ginger Bacterial Wilt in Acidic Continuous Cropping Soil
Source: Front Microbiol. 2020 Nov 30;11:569512. doi: 10.3389/fmicb.2020.569512 (PMC7793916; doi:10.3389/fmicb.2020.569512)

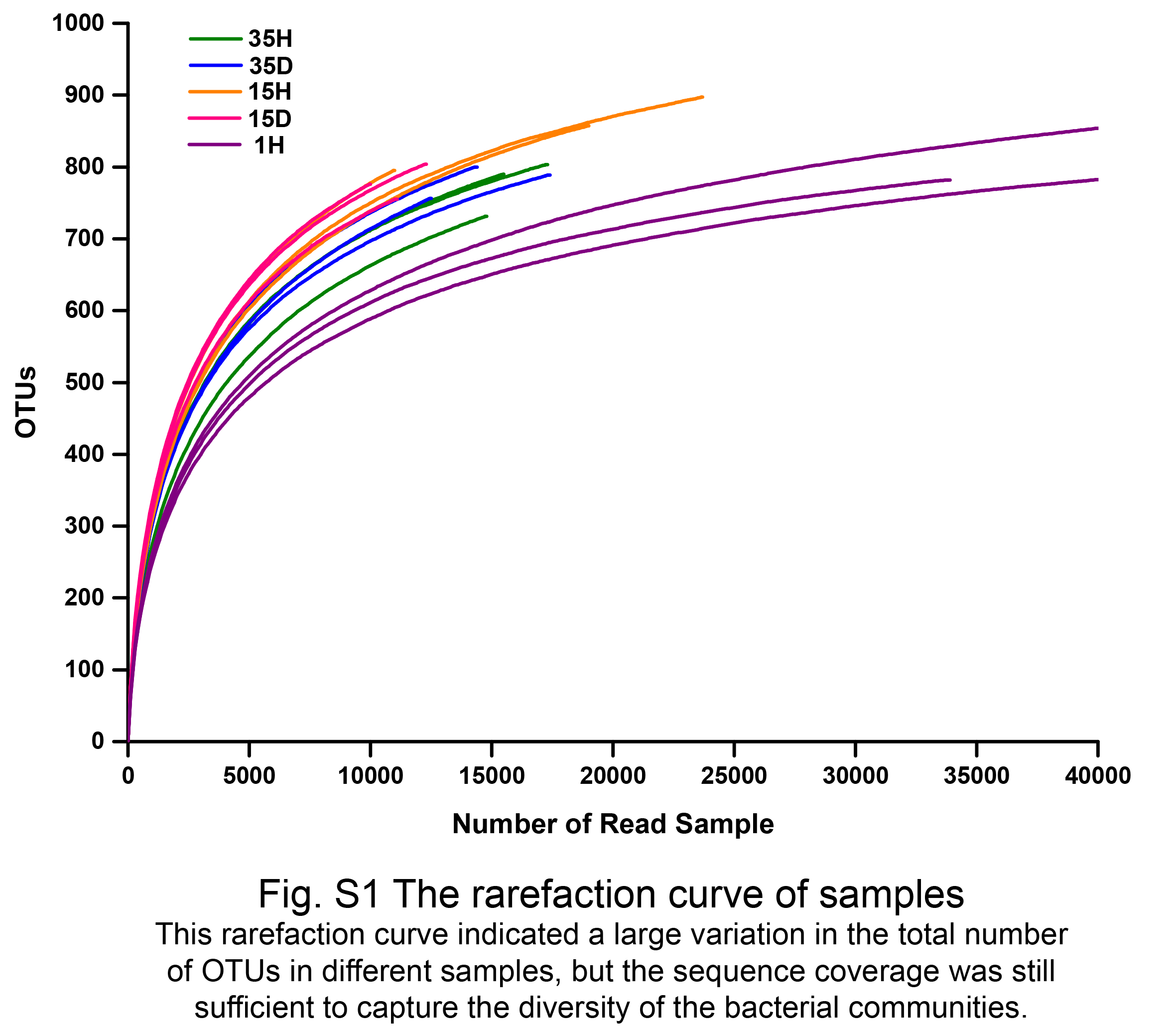

Supplement: Supplementary file 1 [file Image_1.TIF]

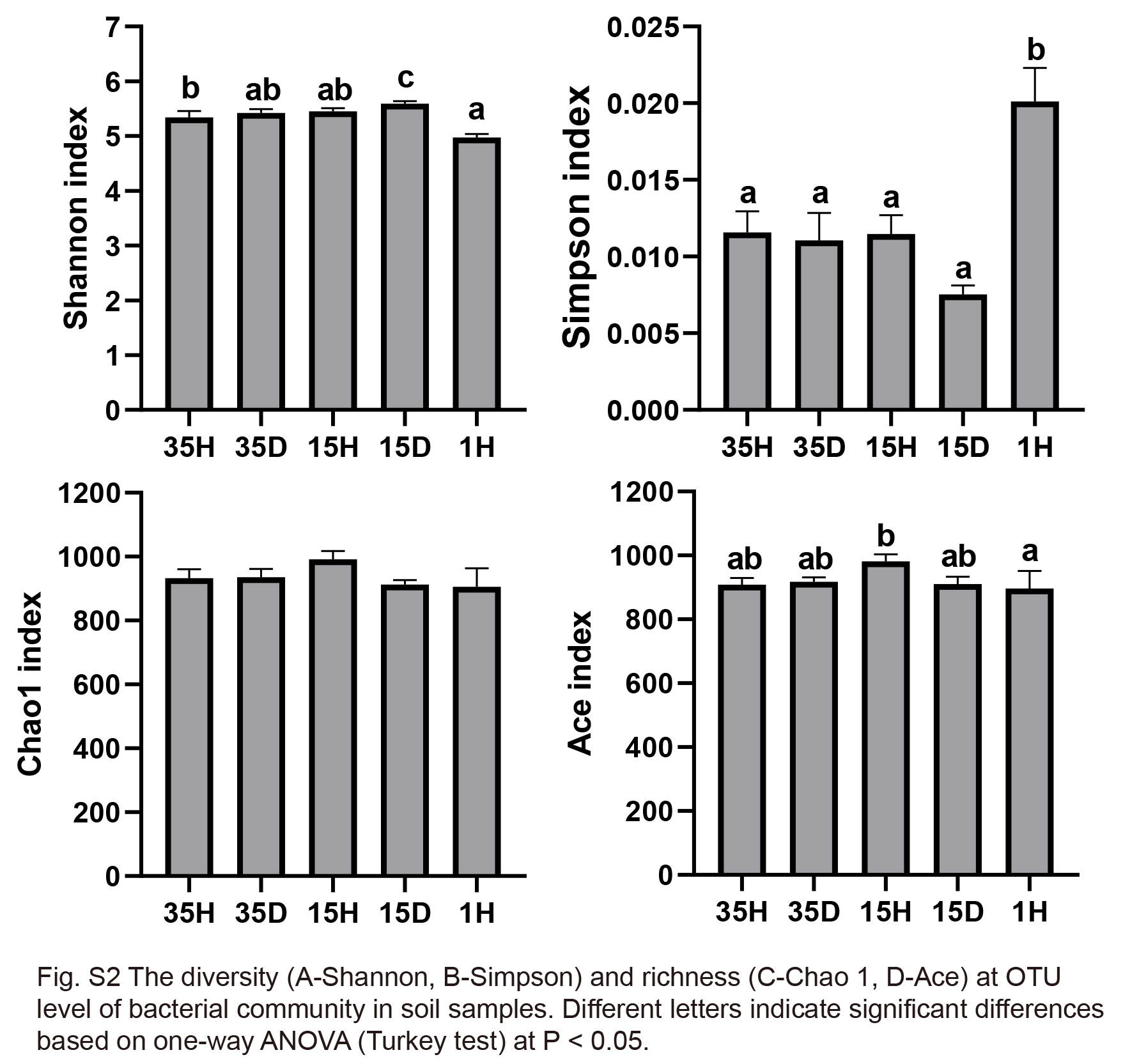

Supplement: Supplementary file 2 [file Image_2.TIF]

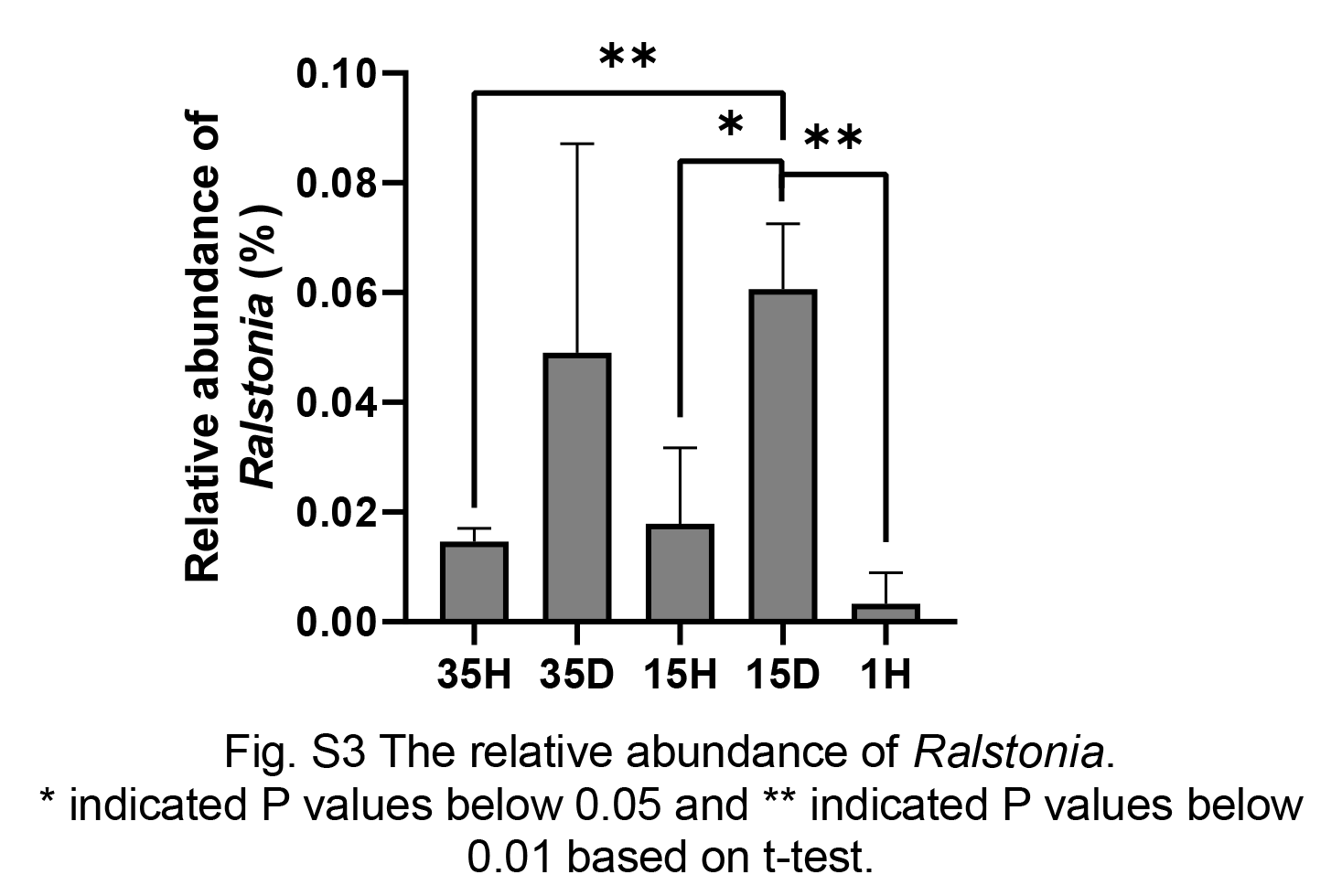

Supplement: Supplementary file 3 [file Image_3.TIF]
