# Supplementary material for: Plant Growth Promoting Rhizobacteria Alleviate Aluminum Toxicity and Ginger Bacterial Wilt in Acidic Continuous Cropping Soil
Source: Front Microbiol. 2020 Nov 30;11:569512. doi: 10.3389/fmicb.2020.569512 (PMC7793916; doi:10.3389/fmicb.2020.569512)
Supplement: Supplementary file 4 [file Table_1.DOCX]

Table S1 The basic chemical properties of each soil sample

| Treatment | Soil type | Organic matter (g/kg) | | Total nitrogen (g/kg) | Total phosphorus (g/kg) | Total potassium (g/kg) | Available phosphorus (mg/kg) | Available potassium (mg/kg) | Available copper（mg/kg） |
| --- | --- | --- | --- | --- | --- | --- | --- | --- | --- |
| 35H | Clay soil | 12.71 | 0.919 | | 1.082 | 9.40 | 193.39 | 203.33 | 5.16 |
| 35D | Clay soil | 20.77 | 0.851 | | 1.050 | 11.06 | 175.42 | 195.00 | 3.65 |
| 15H | Clay soil | 18.98 | 1.193 | | 0.877 | 12.33 | 114.13 | 295.00 | 5.06 |
| 15D | Clay soil | 17.26 | 0.877 | | 0.708 | 7.80 | 84.54 | 178.33 | 4.68 |
| 1H | Clay soil | 24.33 | 1.749 | | 1.066 | 20.36 | 72.00 | 615.00 | 3.96 |
