# Supplementary material for: Plant Growth Promoting Rhizobacteria Alleviate Aluminum Toxicity and Ginger Bacterial Wilt in Acidic Continuous Cropping Soil
Source: Front Microbiol. 2020 Nov 30;11:569512. doi: 10.3389/fmicb.2020.569512 (PMC7793916; doi:10.3389/fmicb.2020.569512)
Supplement: Supplementary file 5 [file Table_2.DOCX]

Table S2 Correlation coefficients (Spearman) between aluminum toxicity index and PGPRs

| PGPR | ExAl | Ca^2+^/Al^3^ | Mg^2+^/Al^3+^ | Ca^2+^/  (Ca^2+^+Fe^3+^+Al^3+^) |
| --- | --- | --- | --- | --- |
| *Pseudomonas* | 0.333 | -0.792** | -0.770** | -0.792** |
| *Bacillus* | 0.670** | -0.789** | -0.775** | -0.789** |
| *Paenibacillus* | 0.009 | 0.154 | 0.136 | 0.154 |
| *Streptomyces* | -0.086 | -0.168 | -0.071 | -0.168 |
| *Arthrobacter* | 0.427 | -0.689** | -0.629* | -0.689** |
| *Serratia* | 0.191 | -0.804** | -0.706** | -0.804** |
| *Flavobacterium* | -0.295 | -0.198 | -0.189 | -0.198 |

Note: * indicated *P* values below 0.05, ** indicated *P* values below 0.01.
